# Case Report: Detection of *Treponema phagedenis* in cerebrospinal fluid of a neurosyphilis patient by metagenomic next-generation sequencing

**DOI:** 10.3389/fcimb.2023.1218049

**Published:** 2023-08-29

**Authors:** Chenyan Yuan, Wei Xu, Chenggui Zhao, Wei Gao, Guoqiu Wu

**Affiliations:** ^1^Center of Clinical Laboratory Medicine, Zhongda Hospital, Southeast University, Nanjing, Jiangsu, China; ^2^Department of Laboratory Medicine, Medical School of Southeast University, Nanjing, Jiangsu, China; ^3^Jiangsu Provincial Key Laboratory of Critical Care Medicine, Southeast University, Nanjing, Jiangsu, China

**Keywords:** *Treponema phagedenis*, cerebrospinal fluid, neurosyphilis, case report, etiological tests

## Abstract

*Treponema phagedenis*, a human commensal spirochete, has been reported world-wide as a key factor in the pathogenesis of bovine digital dermatitis. Here we report a case of *T. phagedenis* sequence detection in the cerebrospinal fluid (CSF) of a patient. The patient was diagnosed with neurosyphilis, and *T. phagedenis* was detected as the only microorganism in his CSF by metagenomic sequencing. The patient went through a round of penicillin therapy previously (2.4 million units of Benzathine Penicillin intramuscularly once a week for three weeks) that did not resolve the symptoms; after the diagnosis of neurosyphilis he was treated with Penicillin G Sodium 4.0 million units q4h intravenous for 14 days then his symptoms resolved. To the best of our knowledge, *T. phagedenis* has never been reported to be detected in a human’s CSF before. This was also the first time it was detected by metagenomic next-generation sequencing. We propose that more etiological tests should be performed including culture and sequencing for more patients with syphilis, which will contribute to a deeper understanding of the pathogenicity of the spirochete.

## 1 Introduction

*Treponema* are Gram-negative, helically coiled, strictly anaerobic, or microaerophilic cells (Radolf, 1996). Fastidious growth requirements and the fragile nature of the species in the *Treponema* genus complicate the determination of taxonomic relationships among them and make their study difficult (Fouad, 2016; Everall and Leonor, 2017). *Treponema* have been frequently reported in humans, pigs, and cattle’s oral, rectal, and genital tracts with diverse roles in a variety of niches (Paster et al., 1998; Evans et al., 2012). There are 28 validated species in the genus according to the List of Prokaryotic Names with Standing in Nomenclature (Parte et al., 2020), including both commensal and pathogenic spirochetes. Pathogenic *Treponema* are host-associated and implicated in many diseases of humans and animals with a complicated relationship with disease etiology. Four species/subspecies of the genus are the agents of human invasive treponematoses: *Treponema pallidum* subsp. *pallidum* (syphilis), T*reponema pallidum* subsp*. pertenue* (endemic syphilis), *Treponema pallidum* subsp*. pertenue* (yaws) and *Treponema carateum* (pinta) (Miao and Fieldsteel, 1978; Miao and Fieldsteel, 1980). *Treponema medium* and *Treponema denticola* are implicated in the polymicrobial etiologies of human and canine periodontal disease (Umemoto et al., 1997; Dashper et al., 2011). Non-pathogenic *Treponema* may be part of the normal flora of the intestinal tract, the oral cavity, or the genital tract of humans and animals. For example, *Treponema rectale* has been reported to be isolated from the bovine rectum (Staton et al., 2017), while *Treponema peruense* is a commensal spirochete isolated from human feces (Belkhou et al., 2021), and *Treponema ruminis* has been isolated from the rumen of cows (Newbrook et al., 2017). *T. phagedenis* used to be isolated as well as other spirochetes from human skin lesions and the genital tract during the original pursuit of the syphilis agent (Noguchi, 1912; Fukunaga et al., 1992). *T.* phagedenis was well studied by comparison with *T. pallidum* and was considered non-pathogenic for human beings (Moskophidis and Müller, 1984; Thomas et al., 1988). Although the species ‘*Treponema phagedenis*’ has been known for more than 100 years, the name was not validly published until 2020. Kuhnert et al. proposed the valid species nomenclature on the base of phenotypic and genotypic features of *T. phagedenis* isolates from bovine and humans (Kuhnert et al., 2020). Their results also indicated that the sequence of *T. phagedenis* genomes originating from bovine and humans were highly conserved; only slight variations can distinguish isolates from different host sources. As an accepted key agent in the pathogenesis of bovine digital dermatitis, a widespread infectious foot condition of economic and animal welfare importance, *T. phagedenis* has been reported many times over the years (Rosander et al., 2011; Espiritu et al., 2020; Khemgaew et al., 2021). *T. phagedenis* has never been detected from the central nervous system (CNS) (using any method). We observed the first detection of *T. phagedenis* from a patient’s cerebrospinal fluid, and this was also the first time that the pathogen was detected by metagenomic next-generation sequencing (mNGS). The patient was clinically diagnosed with neurosyphilis (tabes dorsalis). *T. phagedenis* was the only microorganism identified in the patient’s CSF by mNGS.

## 2 Case report

The patient was a 62-year-old man with a 1.5 year history of numbness in both lower limbs and a positive serological test result for syphilis for six months. In March 2021, he experienced anesthesia and a sore sensation in both lower limbs without any apparent cause, and the feeling worsened after bending and long walks. After 8 months, he felt weakness in both lower legs after prolonged walking. After 12 months, he attended our hospital for gradually increasing difficulty in walking and pain in both lower limbs. He was diagnosed with lumbar spinal stenosis and lumbar disc herniation by the orthopedics department. In a routine examination, his blood tests for rapid plasma reagin (RPR) test (1:4) as well as the *T. pallidum* particle agglutination assay (TPPA) were positive in serum. The *human immunodeficiency virus* (HIV) antibodies were negative (routine commercial assay). Lumbar puncture was recommended to him to exclude neurosyphilis, but he was discharged and did not follow the medical advice of going to dermatology for further treatment. After 17 months, he received 2.4 million units/week intramuscular injections of Benzathine Penicillin in his local hospital for three weeks with no significant improvement.

**Figure 1:**
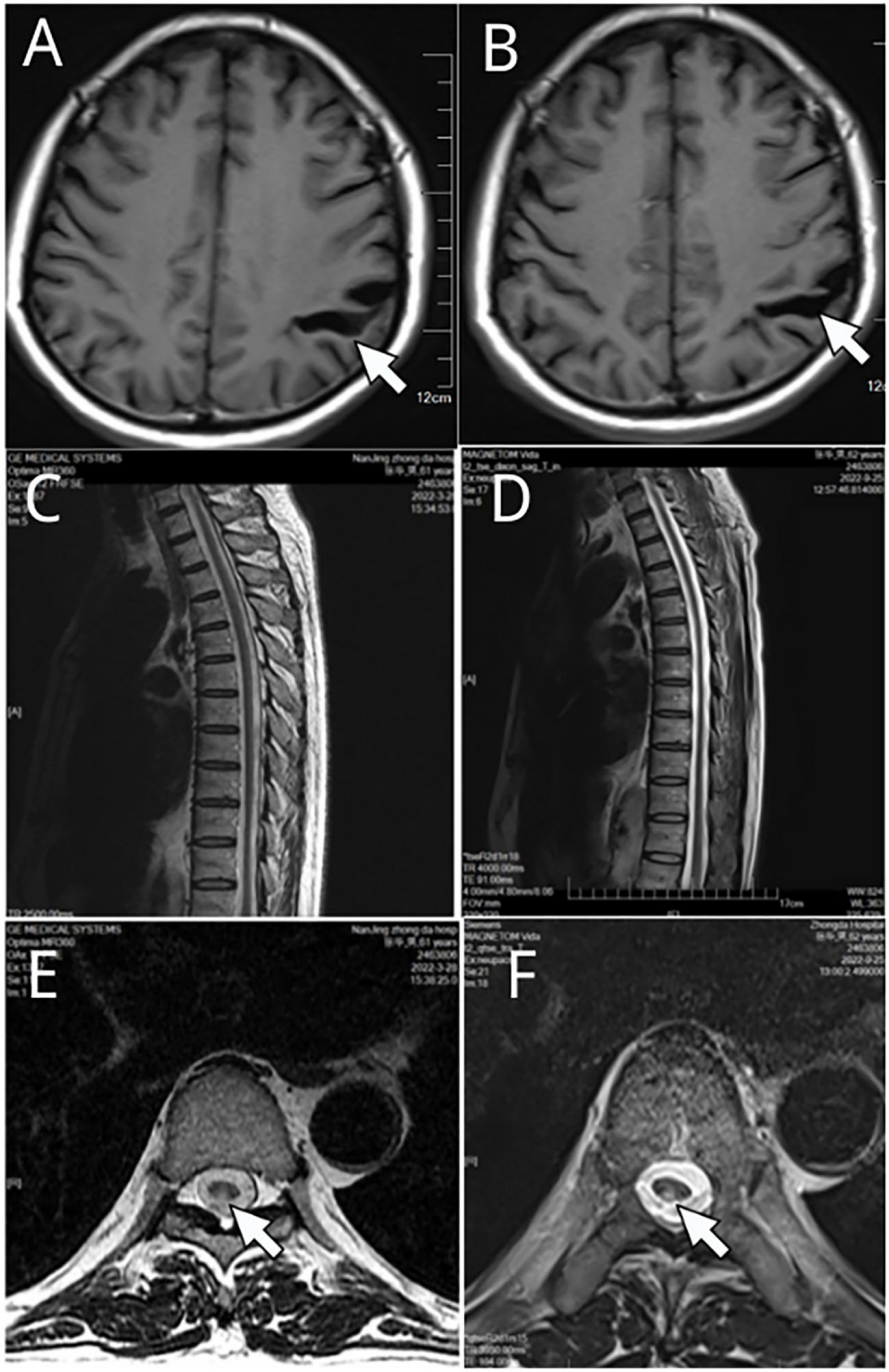
Nuclear magnetic resonance imaging (MRI) of the thoracic spine and cranial brain. **(A, C, E)** were results in Mar 2022, and **(B, D, F)** were examination results in Sep. 2022. A widening of the left parietal sulcus was observed in **(A, B)** (white arrow indicated). Slightly higher signal at the level of the posterior cord of the spinal cord **(E, F)** white arrow indicated) in the T3-6 segment **(C, D)** are shown.

18 months later, he visited our hospital again. Physical examination revealed reduced pain sensation in his lower extremities and no obvious skin rash was observed. On motor examination he had positive results in the heel-knee-tibia test and Romberg’s test. Laboratory investigation showed positive serum results of RPR test (1:2) and TPPA test, normal results of antinuclear antibody, rheumatoid factor, and serum tumor markers. Nuclear magnetic resonance imaging (MRI) of the thoracic spine and cranial brain showed a slightly higher signal at the level of the posterior cord of the spinal cord in the T3-6 segment and a widening of the left parietal sulcus, which had little change compared with the results of examination 6 months previously (**Figure 1**).

On day 3 of the admission, the patient received a lumbar puncture and the pressure of CSF was 115 mmH_2_O. CSF laboratory examination indicated elevated levels of protein (738.5 mg/L) and IgG index (0.89), positive results of RPR test (1:1) and TPPA test. The level of glucose, chlorides, and cells of CSF was normal. Metagenomic next-generation sequencing (MGISEQ 2000 platform, MGI Tech CO., Ltd, ShenZhen, China) of the CSF was done. Sequencing of the sample generated a total of 45469988 reads, of which 79.27% were human DNA sequences. High-quality sequencing data were obtained by filtering out low-quality reads using FastP. To eliminate human genome contamination, the reads were aligned to the GRch38 human reference genome using the Burrows-Wheeler-Alignment Tool (BWA). Among the remaining 2481482 reads, 2067412 reads were mapped to the *T. phagedenis* genome (CP054692.1 *T. phagedenis* strain KS 1 chromosome complete genome, BWA) and the number of reads stringently mapped (SMRN) to the genome in species level was 1988852. Almost all of the remaining sequence reads mapped to some environmental bacterial genomes. However no nucleic acid sequence of the reads was mapped to the *T. pallidum* genome, any viral genomes, any fungi genomes, and any parasite genomes. *T. phagedenis* was detected as the only microorganism in the CSF by metagenomic sequencing and the genome coverage rate of which was 88.53% (3329235 bp/3760559bp). The data presented in the study are deposited in the China National Center for Bioinformation (CNCB) repository, accession number CRA011061.

**Figure 2:**
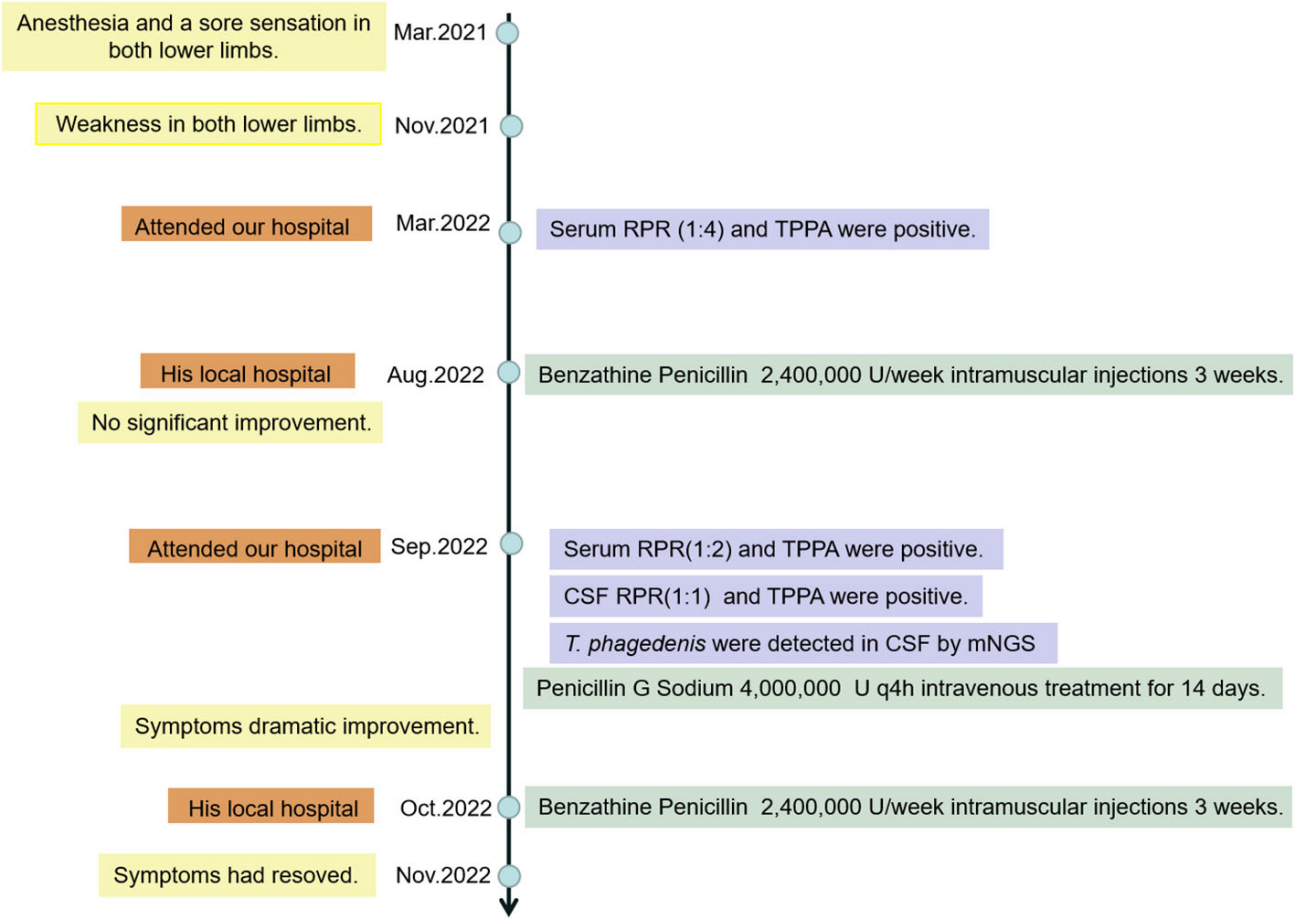
Timeline for patient treatment.

The patient had been given Penicillin G Sodium 4.0 million units q4h intravenous treatment for 14 days and his symptoms showed dramatic improvement. The route of transmission of the patient’s infection remains unclear. We only know that the patient’s wife had positive results of the serum RPR test (1:2) and TPPA test. After treatment, the patient was discharged from the hospital and was given Benzathine Penicillin 2.4 million units intramuscularly once a week for three weeks. The patient was followed up for one month and his symptoms had resolved. The clinical process of diagnosis and treatment of the patient is shown in the flow chart of the timeline (**Figure 2**).

## 3 Discussion

**Figure 3:**
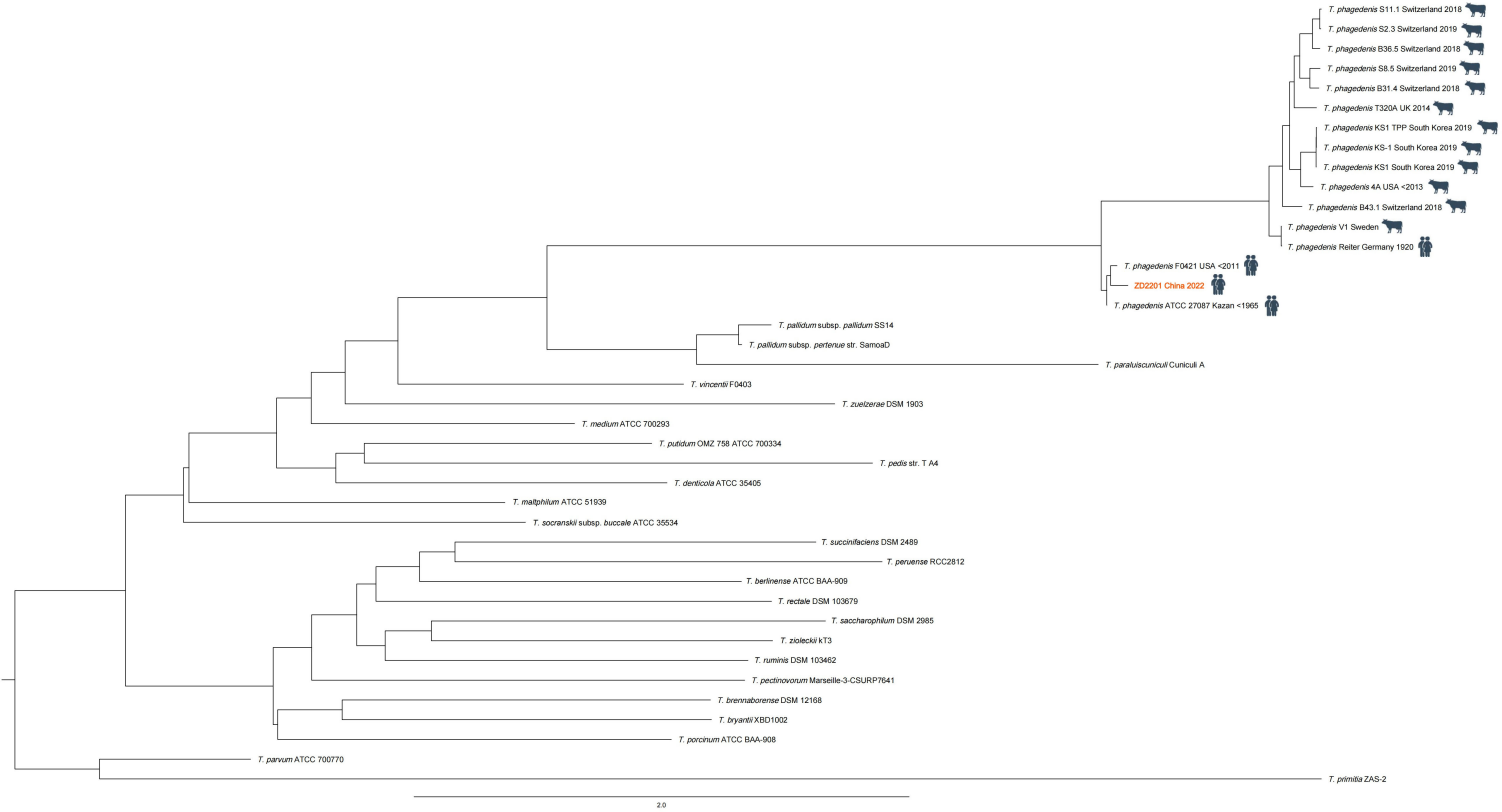
Phylogenetic tree constructed (by KSNP3) based on the whole genome sequencing data of the genus *Treponema. Treponema phagedenis* F0421 (NZ_AEFH00000000.1) was the closest relative of the *T.phagedenis* ZD2201 (the red font).

In this case the nucleotide reads of *T. phagedenis* in the sequencing data were assembled into 3012 scaffolds (*T. phagedenis* ZD2201, CNCB available in **Supplementary Materials 1****,** **2**) and the phylogenetic tree was constructed by KSNP3 (Gardner et al., 2015) based on the reference genome sequencing data of the genus *Treponema* available in the NCBI (National Center for Biotechnology Information) database (**Figure 3**). The phylogenetic tree revealed a clear separation among the *Treponema* species, with all strains of *T. phagedenis* forming a distinct lineage. The human isolates of *T. phagedenis* seemed to form a cluster on the phylogenetic tree that was separated from the bovine isolates. The result was in agreement with that of Clegg et al. (Clegg et al., 2016), who reported that *T. phagedenis* human isolates were as diverse as animal strains and were separated from the animal strains by Multi-Locus Sequence Typing (MLST) analysis.

Unfortunately, since the patient’s CSF was not cultured anaerobically and there was no opportunity to have a second lumbar puncture it was not possible to culture *T. phagedenis* in this case and the pathogenicity of *T. phagedenis* cannot be determined. Due to the fastidiousness and almost non-culturable characteristics of *T. pallidum* subsp. *pallidum* (without tissue culture cells) (Edmondson and Norris, 2021), since there are reports now of culture, albeit difficult and not suitable for routine lab testing, etiological diagnosis of syphilis relies on syphilis serology tests, dark field microscopic examination for spirochetes and molecular biology detection techniques. The term “neurosyphilis” refers to infection of the CNS by *T. pallidum*, subsp. *pallidum*. Neurosyphilis can occur at any time after initial infection. *T. phagedenis* strain ATCC 27087 was isolated from a case of syphilis (Kuhnert et al., 2020) which may support the rare association of *T. phagedenis* and syphilis-like diseases shown by the current study. The laboratory diagnosis of neurosyphilis is based on abnormal results of serum and CSF serologic tests and on elevations in the CSF white-cell count and protein level (Ropper, 2019). However, antigen cross reactivity between species of *Treponema* has been reported. In 1990, Luther and colleagues reported that there was antigenic cross-reactivity between *Borrelia burgdorferi, Borrelia recurrentis, T. pallidum*, and *T. phagedenis*, although none of the borrelial immune sera tested were reactive in the *Treponema pallidum* Hemagglutination Assay (TPHA), anti-*T. phagedenis* immune serum showed a weak reaction in the TPHA (Luther and Moskophidis, 1990). In 1991, Yelton et al. found that *T. phagedenis* encodes and expresses homologues of the *T. pallidum* TmpA and TmpB proteins (Yelton et al., 1991).

Of course, despite the detection of *T. phagedenis* sequences in the patient’s CSF, the case has many limitations, and whether *T. phagedenis* is the key agent of the patient’s CNS infection has not been elucidated, and the pathogenicity of *T. phagedenis* remains unclear. Etiological tests including culture and sequencing are recommended for more patients with syphilis.

## Data availability statement

The datasets presented in this study can be found in online repositories. The names of the repository/repositories and accession number(s) can be found in the article/**Supplementary Material**.

## Ethics statement

Written informed consent was obtained from the individual(s) for the publication of any potentially identifiable images or data included in this article. Written informed consent was obtained from the participant/patient(s) for the publication of this case report.

## Author contributions

WX collected and analyzed the data. CZ and WG took clinical care of the patient and organized the patient’s information. GW provided supervision. CY drafted the manuscript. All authors approved the final version.
