# Supplementary material for: Case Report: Detection of Treponema phagedenis in cerebrospinal fluid of a neurosyphilis patient by metagenomic next-generation sequencing
Source: Front Cell Infect Microbiol. 2023 Aug 29;13:1218049. doi: 10.3389/fcimb.2023.1218049 (PMC10497860; doi:10.3389/fcimb.2023.1218049)
Supplement: Supplementary file 2 [file Table_1.doc]

Sequencing methods:

mNGS was performed upon sample acquisition. A total of nucleic acid was extracted from 300 μl of CSF using a QIAamp DNA Micro Kit (Qiagen, Germantown, MD, USA). DNA sequencing libraries were constructed using a CoolMPS High-throughput Sequencing Set (MGI, Wuhan, China), and the library's quality was assessed using a Qubit Nanodrop instrument. Qualified libraries were amplified, quantitated, and subjected to single-end sequencing on a BGISEQ 2000 sequencer (MGI, Shenzhen, China). High-quality sequencing data were obtained by filtering out low-quality reads using FastP, resulting in high-quality short sequences.

To remove human genomes, the short reads were mapped to the GRch38 reference genome using BWA. De novo assembly was performed using SPAdes, generating 1000 scaffolds. All scaffolds were then mapped to the nr database using BLAST with a cutoff value of 1e-5. Only sequences that mapped to the Treponema genome were saved to obtain the assembly of T. phagedenis.
