# Supplementary material for: Case Report: Detection of Treponema phagedenis in cerebrospinal fluid of a neurosyphilis patient by metagenomic next-generation sequencing
Source: Front Cell Infect Microbiol. 2023 Aug 29;13:1218049. doi: 10.3389/fcimb.2023.1218049 (PMC10497860; doi:10.3389/fcimb.2023.1218049)
Supplement: Supplementary file 3 [file Table_2.doc]

Treponema phagedenis used to be isolated as well as other spirochetes from the human skin lesions and genital tract during the original pursuit of the syphilis agent. Treponema phagedenis has been well studied by comparison with Treponema pallidum and was considered non-pathogenic for human beings. As an accepted key agent in the pathogenesis of bovine digital dermatitis, a widespread infectious foot condition of economic and animal welfare importance, isolation of Treponema phagedenis has been reported many times over the years. Treponema phagedenis has never been detected from the central nervous system (using any method). This is the first time the pathogen has been detected in human cerebrospinal fluid (CSF), and the first time it has been detected using metagenomic next-generation sequencing (mNGS). The patient was clinically diagnosed with neurosyphilis (tabes dorsalis). Treponema phagedenis was the only microorganism identified in the patient's CSF by mNGS. Etiological tests including culture and sequencing are recommended for more patients with syphilis, which will contribute to a deeper understanding of the pathogenicity of the spirochete.
